# Cases Illustrative of the Beneficial Effects of the Oil of Turpentine

**Published:** 1840-12

**Authors:** John Bennett

**Affiliations:** Late of Newport, Kentucky


Art. II.—*Cases Illustrative of the Beneficial Effects of the Oil of Turpentine.* By Dr. John Bennett, late of Newport, Kentucky.

Hæmorrhoids. In July, 1830, having charge of the health of the troops, at the United States Arsenal in Newport, a recruit who labored under piles of long standing and aggravated character, was attacked with colic. The abdomen was much distented. I ordered him ol. ricini ℥i. combined with ʒij. of oil of turpentine. On my visit the next day, I found him relieved from colic and decidedly better of the piles. The turpentine was continued for a few days, and the patient returned to duty free from the disease.

Since that time I have given the castor oil and turpentine, in that disease with uniform success.

Worms. For several years I have laid aside the usual vermifuges, and given the turpentine exclusively in cases of worms, and it has rarely disappointed my expectations. In the convulsions of children, ascribed to worms, I have given the turpentine and oil with the happiest effects, when no worms passed off. If the convulsions of children arise from some other irritation of the mucous membrane of the stomach or bowels, the turpentine and oil are, so far as my experience goes, the most efficient remedy. Their action is twofold—they soothe the irritated membrane, and at the same time carry off the irritating matter.

Since writing the above, I was called to see a child of Mr. H. It had been attacked with convulsions. Gave a portion of oil and turpentine. The medicine operated well, and it had no return of the fits.

Chronic Cholera Infantum. In July last, I was called to the country, some miles distant, to see several patients laboring under the fever. I was there informed, by a lady, that Cholera Infantum was rife in her neighborhood, and that it was universally cured by the turpentine and oil. In the acute stages of that disease, I have not tried it; but in the chronic form, it has succeeded better than any other remedy, to which I have resorted. I select out of my note book the following, from among a number of similar cases.

July 11th. Called to Mr. D.'s child, aged 18 months, with cholera infantum. Directed strong coffee without sugar or cream, to be given every fifteen or twenty minutes, with an occasional enema of salt and water, and a sinapism to the region of the stomach and bowels. *Visit at night.* Stomach less irritable. Frequent watery discharges from the bowels. If water is taken it is instantly thrown up. ℞. sub. mur. hydr., grs. iij.; sacch. alb. ʒss., mix and divide into twelve powders. One to be given hourly. 12th. Stomach still irritable, thirst great, pulse quick, extremities cold, discharges frequent and watery. Continue the calomel; cold applications to the head, which is hot; drink, slippery elm tea; diet, arrow root. 13th. Vomiting has ceased; discharges from the bowels frequent. Calomel and Dover's powder, in small, repeated portions. 15th. Purging continues. Gave chalk and pulverized cinnamon. 16th. Discharges from the bowels variable in color and consistence. Directed the chalk mixture. *Evening;* some tumefaction of the abdomen ; manifestation of pain. Give hyd. cum. creta and morphine. 17th. Belly tumid ; discharges slimy and bloody. Directed oil and turpentine. 18th. Swelling of the abdomen subsided ; discharges this morning more natural. From this time the convalesence was rapid and my little patient now enjoys excellent health.

Dysentery. About the 1st of July, this disease began to prevail in this place and its vicinity. A number of cases which came under my notice assumed a chronic form, in most of which, I gave the oil and turpentine with unequivocal success.

*Case.* A child of Mr. T.'s was attacked with cholera infantum, which terminated in dysentery, with a tympanitic state of the bowels, emaciation, &c. Gave the oil of turpentine, which afforded relief in a short time.

*Case.* A little son of Maj. T. of Newport, had an attack of dysentery, which was treated by Professor Harrison, of Cincinnati. After he had recovered from that disease, he was attacked with diarrhœa, when he became my patient. The usual remedies were put into requisition, without permanent relief, when I directed a portion of olive oil and oil of turpentine mixed. On my next visit, Mrs. T. informed me that the turpentine had acted like a charm, in removing the disease.

*Case.* A highly respectable lady in Covington was attacked with dysentery in July last. Her disease assumed a chronic character, and was removed by a few portions of the oil and turpentine. In short, I have found no remedy equal to the oil of turpentine, in chronic dysentery ; and I am decidedly of opinion that its effects would be equally beneficial in the acute form of the disease, or in any other diseased action of the mucous membranes.

In Gonorrhœa I have lately given the following preparation, which has removed the disease in less time and more effectually, than the bals. copaiva has ever done in my hands.

℞. Oil of turpentine ℥i.

Refined sugar ℥i.

Powdered gum Arabic ʒij.

Mint water ℥viij. Mix.

Of this, I give a table spoonful three times a day.

In Duodenitis attended with jaundice, I have uniformly succeeded with the turpentine.

*Case.* On the 30th October last, I was requested to prescribe for a young man, who labored under jaundice, with tenderness in the region of the duodenum. Tongue coated in the middle, and red round the edges. I directed him to take a blue pill every night for three nights, to be followed, the next morning, with a dose of oil and turpentine. The three portions removed the disease.

*Case.* I was this day, 14th of November, requested to visit a servant girl of Major T.'s of Newport. She was attacked on the 12th with pain in the epigastrium, and occasional vomiting. Her mistress, supposing she had colic, gave her a portion of calomel and afterwards a dose of castor oil, which procured several stools with little or no relief. She now had violent periodical pain in the epigastric region; great tenderness on pressure; tongue with white fur, red on the edges; pulse full, slow and soft; skin dry; whites of the eyes yellow. Directed calomel and Dover's powder, followed by castor oil and turpentine. 15th. Less pain; the medicine has acted on the bowels, but not freely. Direct a continuation of the medicine. 17th. Mending; free from pain. 20th. Well.

I could enumerate many other cases, proving the utility of turpentine. Perhaps there is no one medicine which can be applied with equal advantage in such a variety of diseases. I have usually combined it with oil, either castor or olive.

*November*, 1838.

